# Association of *TLR4* and *TLR9* gene polymorphisms and haplotypes with cervicitis susceptibility

**DOI:** 10.1371/journal.pone.0220330

**Published:** 2019-07-31

**Authors:** Alex Chauhan, Nilesh Pandey, Ajesh Desai, Nitin Raithatha, Purvi Patel, Yesha Choxi, Rutul Kapadia, Ronak Khandelwal, Neeraj Jain

**Affiliations:** 1 P D Patel Institute of Applied Sciences, Charotar University of Science and Technology (CHARUSAT), Changa, Anand, India; 2 Department of Obstetrics and Gynaecology, GMERS Medical College and Hospital, Ahmedabad, India; 3 Department of Obstetrics and Gynaecology, Pramukh Swami Medical College, Shree Krishna Hospital, Karamsad, India; 4 Department of Obstetrics and Gynaecology, Sir Sayajirao General Hospital and Medical College Baroda, Vadodara, India; Bharathidasan University, INDIA

## Abstract

**Background:**

Cervicitis is one of the major health problems amongst women caused by infection of various pathogens including *Chlamydia trachomatis* (CT), *Neisseria gonorrhoeae* (NG), *Trichomonas vaginalis* (TV) as well as human papillomavirus (HPV), and persistent cervical inflammation is one of the etiologic agents of cervical cancer. Toll-like receptors (TLRs) play an important role in the recognition and subsequent elimination of these pathogens. Variations in the Toll-like receptor genes influence susceptibility to pathogens as well as disease progression independently.

**Methods:**

Ten single nucleotide polymorphisms, five each of *TLR4* and *TLR9* genes were analyzed among 130 cervicitis patients and 150 controls either using polymerase chain reaction-restriction fragment length polymorphism or allele specific-PCR.

**Results:**

*T*. *vaginalis* infection was found at the highest frequency (30.7%) as compared to *C*. *trachomatis* (1.5%), *N*. *gonorrhoeae* (2.3%) and HPV (4.6%) infections in cervicitis patients. *TLR4* rs11536889 CC (age-adjusted OR, 2.469 [95% CI, 1.499 to 4.065]; p < 0.001) and *TLR9* rs187084 TC (age-adjusted OR, 2.165 [95% CI, 1.267–3.699]; p = 0.005) genotypes showed the higher distribution in cervicitis patients compared to controls. In addition, TLR4 rs11536889 C allele was shown to increase the risk of cervicitis (age-adjusted OR, 1.632 [95% CI, 1.132 to 2.352]; p = 0.009) compared to controls. The *TLR4* haplotype GCA (OR, 0.6 [95% CI, 0.38–0.95]; p = 0.0272) and *TLR9* haplotype GTA (OR, 1.99 [95% CI, 1.14–3.48]; p = 0.014) were found to be associated with decreased and increased risk of cervicitis respectively.

**Conclusions:**

*TLR4* and *TLR9* polymorphisms, as well as haplotypes were shown to modulate the cervicitis risk.

## Introduction

Cervicitis, i.e., the inflammation of cervix, is chiefly caused by the infections of *Chlamydia trachomatis* (CT) and *Neisseria gonorrhoeae* (NG). Infections of *Trichomonas vaginalis* (TV) and human papillomavirus (HPV) have also been implicated in the pathogenesis of cervicitis, in addition to other bacterial and viral pathogens [1]. Of these microbes, CT and NG infections lead to pelvic inflammatory disease (PID), endometritis and infertility whereas certain cases of preterm birth and low weight babies are associated with TV infection [2]. Women with cervical inflammation and HPV infection have increased risk of developing high-grade squamous intraepithelial lesions [3,4] as well as cervical cancer [5–10]. Moreover, chronic inflammation is considered as a contributory factor in the development of various cancers including cervical cancer.

Upon infection, these pathogens activate the Toll-like Receptors (TLRs) which are one of the types of pattern recognition receptors (PRRs) present on the host innate immune cells that recognizes pathogen-associated molecular patterns (PAMPs) and stimulates antigen-specific acquired immunity for pathogen elimination [11–13]. Ten functional TLRs are known in humans designated as TLR1 to TLR10. The recognition of CT, NG, TV and HPV infections are mediated either by TLR4 or TLR9 [14–16]. The transmembrane TLR4 recognizes lipopolysaccharides (LPS) present in bacterial cell wall as well as viral proteins whereas intracellular TLR9 recognizes unmethylated CpG DNA of various pathogens [13,17,18].

*TLR* polymorphisms have also been associated with increased susceptibility to a wide range of bacterial and viral infections [19–25]. However, limited studies have investigated the role of *TLR* single nucleotide polymorphisms (SNPs) in CT, NG [19,26–29], TV [30] and HPV [31–33] infection associated complications but none on cervicitis.

Among women with PID, *TLR4* polymorphism showed association with increased CT as well as NG infection [26,27]. *TLR4* and *TLR9* SNPs have also been reported to be related to higher risk of tubal pathology following CT infection [29]. *TLR4* polymorphisms Asp299Gly and Thr399Ile were associated with a decreased incidence CT and NG in tubal factor infertility patients [19]. Chen et al. (2013) observed a marginal association of the *TLR4* SNP with TV infected prostate cancer [30]. No association of *TLR9* promoter polymorphism was found either with HPV clearance or persistence in healthy women [32]; however, another *TLR9* coding region non-synonymous polymorphism revealed a higher risk of cervical carcinogenesis in the presence of HPV16 infection [31,33].

Moreover, though non-synonymous and promoter regions SNPs offer an appropriate relevance, intronic and synonymous polymorphisms are seldom appreciated for their role in genetic association studies. However, the later type of SNPs do confer disease susceptibility due to their presence in alternative splicing, trans-splicing, and other regulatory elements [34–38]. Thus, we included ten SNPs in our analysis, five of each *TLR4* and *TLR9* gene, covering the untranslated regions, intronic, synonymous and non-synonymous polymorphisms.

As a whole, considering the importance of *TLR* polymorphisms with increased susceptibility to various infections and disease development, and the lacuna of reports on cervicitis, the present was designed to investigate the role of certain common *TLR4* and *TLR9* SNPs of different gene regions to *C*. *trachomatis*, *N*. *gonorrhoeae*, *T*. *vaginalis*, HPV infections, and cervicitis.

## Materials and methods

### Study subjects

Two hundred and eighty subjects attending gynaecologic OPD of Shree Krishna Hospital, Karamsad, Anand and Sir Sayajirao General Hospital and Medical College, Vadodara, India participated in the study. Of these, 130 were diagnosed with cervicitis while rest were age-matched healthy controls. The patients’ samples were either in the form of cervical biopsies or smears while controls were normal cervical smears provided by healthy subjects attending gynaecologic OPD for reasons other than cervicitis. The inclusion criteria included clinical diagnosis followed by cyto/histologic confirmation while sample collection was avoided from subjects undergoing menstruation. The study subjects were recruited from 2012 to 2017. The study was approved by the Institutional Review Board, Ashok and Rita Patel Institute of Physiotherapy, CHARUSAT, Changa, Anand (CIP/IRB/13/16); Institutional Ethics committee, HP Patel Centre for Medical Care and Education, Karamsad (HMPCMCE/HREC/344/11) and Institutional Ethics Committee for Human Research (IECHR) Medical College and SSG Hospital, Vadodara (ECR/85/Inst/GJ/2013), India. All the participants were explained the importance of the study in the vernacular language. An information sheet written in Gujarati as well as English was provided to them and a written consent was obtained from all the study subjects. In the present study, 280 participants were recruited with an allocation ratio of 1.15 (N2/N1), which was sufficient as per the calculation by G*Power v3.1 that revealed a sample size of 275 participants is required to achieve a statistical power of 0.8 at 95% CI.

### DNA extraction

DNA isolation from cervical biopsies/ smears that were collected in the chilled phosphate buffered saline was carried out using the standard phenol-chloroform extraction method [39]. Whenever a sample with a less amount of biopsy/smear was obtained, for example biopsies: weighing ≤10mg and/ or smears represented by a pellet size of ≤5mm obtained upon centrifugation, a spin-column-based DNA isolation kit was utilized as per the manufacturer’s instructions (Macherey-Nagel, Germany). Quality and quantity of DNA was estimated using ethidium bromide-stained 1% agarose gel on a GelDoc system (BioRad, USA) as well as a NanoDrop 2000 (Thermofisher, USA).

## Pathogen detection

Real-time PCR detection of CT, NG, and TV was performed using AmpliSens *T*. *vaginalis* / *N*. *gonorrhoeae* / *C*. *trachomatis*-MULTIPRIME-FRT PCR kit (Ecoli, Slovakia) according to the manufacturer’s instructions across all the samples. HPVs were detected using SYBR Premix Ex Taq II (Tli RNaseH Plus) kit (Takara, Japan) using Real-time PCR. For the detection of HPV, samples were first subjected to HPV consensus PCR using Gp5+/Gp6+ primers [40] followed by detection of HPV16 and 18 using type-specific primers [41]. Typically, a 20μl real-time PCR mix comprised of 1X SYBR Premix Ex Taq (Tli RNAse H Plus), 0.2μM of each forward primer and reverse primer, 1X ROX reference Dye II and 25ng of template DNA. The positive controls for hrHPV types 16 and 18 were obtained as a part of participation in Global HPV Proficiency study, Equalis, Uppsala, Sweden. *β-globin* gene served as an internal control while in the negative control, DNA was replaced with PCR grade nuclease-free water. The Real-time PCR was performed on 7500 Real-Time PCR system (Applied Biosystems, USA).

For the HPV detection by Gp5+/ Gp6+ primers touch down PCR thermal cycling condition was utilized that included initial denaturation at 95°C for 1 min followed by 16 cycles of denaturation at 95°C for 20 s, annealing at 55°C for 30 s (with a decrement of 1°C in each subsequent cycle) and extension at 72°C for 30 s. This thermal profile was further continued for 34 cycles at an annealing temperature of 40°C. HPV16 and 18 were amplified using following thermal cycling condition: initial denaturation at 95°C for 1 min followed by 40 cycles of denaturation at 95°C for 15 s, annealing at 55°C for 30 s and extension at 72°C for 30 s. All the reactions were performed in duplicates that also included positive and negative controls. Each run comprised of both amplification and melt curve stages.

### SNP analysis

Ten SNPs, five each of *TLR4* (rs4986791, rs4986790, rs10759931, rs11536889, rs1927911) and *TLR9* (rs5743844, rs187084, rs5743836, rs352140, rs352139) genes were analysed either using PCR-RFLP or AS-PCR. Eight out of ten SNPs that were selected had minor allele frequency (MAF) >0.05 (S2 Table). On the other hand, *TLR4* rs4986791 as well as *TLR9* rs5743844 SNPs though have MAF <0.05, were selected as these polymorphisms have previously been shown to be associated gastric cancer (*TLR4*) and CpG oligonucleotide hyporesponsiveness (*TLR9*) [42,43]. Information associated with SNPs such as nucleotide/ codon/ amino acid change and the location is mentioned in S1 Table. Primers specific for each SNP, thermal profile, amplicon size as well as associated restriction enzyme, digested product, genotype and mode of visualization is given in S2 and S3 Tables. A typical reaction of 25μl contained 50–100 ng genomic DNA, 0.1mM dNTP mix, 0.1μM of each oligonucleotide primer and 0.8U Taq DNA polymerase (Kapabiosystems, USA). All the reactions were performed on an MJ Mini thermal cycler (BioRad, USA). *TLR9* rs352139 polymorphism was genotyped using AS-PCR while the PCR amplicons of rest of the SNPs were subjected to restriction digestion with their respective enzymes as mentioned in S2 Table. PCR amplicons and digested PCR products were analyzed either on an agarose or on an acrylamide gel under a gel documentation system (BioRad, USA) (S3 Table). Restriction enzymes were procured from New England BioLabs, USA.

### Statistical analysis

All the statistical analyses were performed on SPSS v24, USA. The levels of significance were two-sided and considered significant if the p-values were less than 0.05. The χ^2^ goodness of fit analysis was performed to determine the deviation from Hardy-Weinberg equilibrium among controls. Pearson’s chi-square test was used to determine the differences in the genotype or allele frequencies between the control and the patient groups. Fisher’s exact test was performed to estimate the genotypic or allelic associations. Logistic regression was applied to calculate the age-adjusted odds ratios (OR). The significance of each genotype/ allele was derived using dominant model where the wildtype genotype/allele was considered as a reference.

### Haplotype determination

Pairwise linkage disequilibrium (LD), D', r^2^ as well as LD structure was determined using Haploview v4.2. The D’ confidence interval algorithm created by Gabriel et al., (2002) was used to assign haplotype blocks [44]. Additionally, the haplotype block structure was also generated using Locusview v2.0. To determine the difference in the haplotype frequencies between the cases and control population, global test was performed, and odds ratios were calculated using FAMHAP software v19.

## Results

### Demography and prevalence of pathogens

All 130 cervicitis patients (mean ± SD age, 36.8 ± 10.9 years) and 150 control subjects (mean ± SD age, 34.8 ± 11.8 years) were comparable in age (*p =* 0.142). One hundred seventeen (90%) patients were homemakers while rest were working women. Among normal controls 94 (62.7%) were homemakers, 34 (22.7%) were students while rest were working women and none of them had any previous history of sexually transmitted infections. The prevalence of CT, NG, and TV was found in 2 (1.5%), 3 (2.3%) and 40 (30.7%) cervicitis patients respectively. Moreover, 3 (2%) control subjects also showed the presence of TV. Six (4.6%) cervicitis cases and two controls (1.3%) were detected positive for HPV consensus sequences amongst which HPV16 was detected in 3 (2.3%) patients and in one (0.6%) control. Rest of the three HPV consensus positive cervicitis cases were negative for both HPV16 and 18. Moreover, HPV18 was not detected in any of the subjects. None of the pathogens was found to be coexisting with another pathogen. The frequency of all the pathogens among cervicitis patients is listed in Table 1.

**Table 1:** Prevalence of *T*. *vaginalis*, *N*. *gonorrhoeae*, *C*. *trachomatis* and HPV in cervicitis patients.

*C*. *trachomatis*	N. *gonorrhoeae*	*T*. *vaginalis*	HPV
2 (15.3%)	3 (2.3%)	40 (30.4%)	6 (4.6%)

### Genetic analysis

The genotype frequencies of *TLR4* and *TLR9* SNPs within the control population were in agreement with the Hardy-Weinberg equilibrium except for *TLR4* rs11536889 and rs4986791 as well as *TLR9* rs5743844 polymorphisms. However, the *TLR4* rs11536889 polymorphism was retained for analysis as its homozygous genotype GG was not detected in any of the study subjects, which could be a probable reason for its variance from the Hardy-Weinberg equilibrium.

*TLR4* rs10759931 AG genotype, and rs11536889 GC genotype as well as *TLR9* rs187084 TC genotype, showed a significant difference in their frequency distribution among cases and controls. The cervicitis patients were less likely to carry *TLR4* rs10759931 AG genotype as compared to control population (age-adjusted OR, 0.418 [95% CI, 0.220–0.794]; *p =* 0.008). Contrasting results were obtained for *TLR4* rs11536889 polymorphism, where the CC genotype was found at a higher frequency in patients as compared to controls (age-adjusted OR, 2.469 [95% CI, 1.499–4.065]; *p* < 0.001). Furthermore, the C allele of the same polymorphism was also found to be statistically higher in patients as compared to controls (age-adjusted OR, 1.632 [95% CI, 1.132 to 2.352]; *p =* 0.009). On the other hand, *TLR9* rs187084 TC genotype in cases was found at a statistically higher frequency as compared to control subjects (age-adjusted OR, 2.165 [95% CI, 1.267–3.699]; *p =* 0.005). None of the rest *TLR4* or *TLR9* SNPs showed a statistically significant difference between cases and controls. In an intriguing observation, we found single genotype of *TLR4* rs4986791 (Thr399Ile) and *TLR9* rs5743844 (Pro99Leu) SNPs among all the study subjects. Therefore, both of these SNPs were excluded from further analysis. The final genotypes, as well as allele frequencies of the analyzed SNPs, are listed in Table 2.

**Table 2:** Genotype and allele frequency distribution of *TLR4* and *TLR9* gene polymorphisms in cervicitis and control subjects.

*Gene*	SNPs (rsID)	Cases (%)	Controls (%)	*p*-value^a^	*p*-value^b^	MAF	Age-adjusted OR @ 95% CI
***TLR4***	896 A/G (rs4986790)			0.694		0.141	
	AA	93 (71.5)	113 (75.3)		-		Reference
	AG	35 (26.9)	34 (22.7)		0.472		1.223 (0.707 to 2.117)
	GG	2 (1.5)	3 (2.0)		0.821		0.810 (0.132 to 4.967)
	A	221 (85.0)	260 (86.7)		-		Reference
	G	39 (15.0)	40 (13.3)		0.621		1.128 (0.700 to 1.819)
	2688 A/G (rs10759931)			**0.006**		0.434	
	AA	34 (26.2)	24 (16.0)		-		Reference
	AG	46 (35.4)	81 (54.0)		**0.008**		0.418 (0.220 to 0.794)
	GG	50 (38.5)	45 (30.0)		0.498		0.795 (0.410 to 1.541)
	A	114 (43.8)	129 (43.0)		-		Reference
	G	146 (56.2)	171 (57.0)		0.883		0.975 (0.696 to 1.365)
	3725 G/C (rs11536889)			**0.000**		0.329	
	GG	-	-		-		-
	GC	67 (51.5)	109 (72.7)		**-**		Reference
	CC	63 (48.5)	41 (27.3)		**0.000**		2.469 (1.499 to 4.065)
	G	67 (25.8)	109 (36.3)		**-**		Reference
	C	193 (74.2)	191 (63.7)		**0.009**		1.632 (1.132 to 2.352)
	7764 C/T (rs1927911)			0.955		0.22	
	CC	76 (58.5)	90 (60.0)		-		Reference
	CT	50 (38.5)	56 (37.3)		0.794		1.068 (0.654 to 1.744)
	TT	4 (3.1)	4 (2.7)		0.861		1.136 (0.273 to 4.716)
	C	202 (77.7)	236 (78.7)		-		Reference
	T	58 (22.3)	64 (21.3)		0.787		1.057 (0.706 to 1.583)
***TLR9***	-1486 T/C (rs187084)			**0.020**		0.384	
	TT	45 (34.6)	72 (48.0)		-		Reference
	TC	61 (46.9)	48 (32.0)		**0.005**		2.165 (1.267 to 3.699)
	CC	24 (18.5)	30 (20.0)		0.411		1.317 (0.683 to 2.543)
	T	151 (58.1)	192 (64.0)		-		Reference
	C	109 (42.9)	108 (36.0)		0.144		1.291 (0.917 to 1.818)
	-1237 T/C (rs5743836)			0.400		0.136	
	TT	101 (77.7)	106 (70.7)		-		Reference
	TC	28 (21.5)	42 (28.0)		0.169		0.678 (0.389 to 1.180)
	CC	1 (0.8)	2 (1.3)		0.605		0.527 (0.047 to 5.972)
	T	230 (88.5)	254 (84.7)		-		Reference
	C	30 (11.5)	46 (15.3)		0.165		0.704 (0.429 to 1.155)
	2848 G/A (rs352140)			0.450		0.496	
	GG	29 (22.3)	42 (28.0)		-		Reference
	GA	66 (50.8)	75 (50.0)		0.348		1.321 (0.739 to 2.364)
	AA	35 (26.9)	33 (22.0)		0.210		1.538 (0.785 to 3.015)
	G	124 (47.7)	159 (53.0)		-		Reference
	A	136 (52.3)	141 (47.0)		0.209		1.239 (0.887 to 1.730)
	1174 A/G (rs352139)			0.433		0.484	
	AA	28 (21.5)	35 (23.3)		-		Reference
	AG	72 (55.4)	72 (48.0)		0.379		1.310 (0.718 to 2.387)
	GG	30 (23.1)	43 (28.7)		0.756		0.897 (0.452 to 1.779)
	A	128 (49.2)	142 (47.3)		-		Reference
	G	132 (50.8)	158 (52.7)		0.716		0.940 (0.673 to 1.312)

^a^Pearson’s Chi-square test.

^b^Fisher’s exact test. Significant p-values are represented in bold. Minor allele frequencies were calculated using Haploview.

*TLR*, Toll-like receptor; SNP, single nucleotide polymorphism; rsID, reference sequence ID; MAF, minor allele frequency; OR, odds ratio; CI, confidence interval

On comparing TV infected cervicitis patients to controls, a higher frequency of CC genotype (age-adjusted OR, 2.216 [95% CI, 1.076 to 4.560]; *p =* 0.031) of *TLR4* rs11536889 polymorphism was found among TV positive cases as compared to controls (Table 3). Since the frequency of CT, NG and HPV in the study population was statistically low, further analysis of their association with *TLR4* and *TLR9* SNPs was not performed.

**Table 3:** Genotype and allele frequency distribution of *TLR4* rs11536889 polymorphism among TV positive cases and healthy controls.

SNP (rsID)	Cases (%)	Controls (%)	*p*-value^a^	Age-adjusted OR @ 95% CI
*TLR4* 3725 G/C (rs11536889)				
GG	-	-		
GC	18 (42.4)	108 (72.8)	**-**	Reference
CC	22 (57.6)	42 (27.2)	**0.031**	2.216 (1.076 to 4.560)
G	18 (22.5)	108 (36.4)		Reference
C	62 (77.5)	192 (63.6)	0.135	1.517 (0.879 to 2.618)

^a^Fisher’s exact test. Significant *p*-values are represented in bold.

*TLR*, Toll-like receptor; SNP, single nucleotide polymorphism; rsID, reference sequence ID; OR, odds ratio; CI, confidence interval; *TLR*, Toll-like receptor

### Haplotype analysis

**Fig 1:**
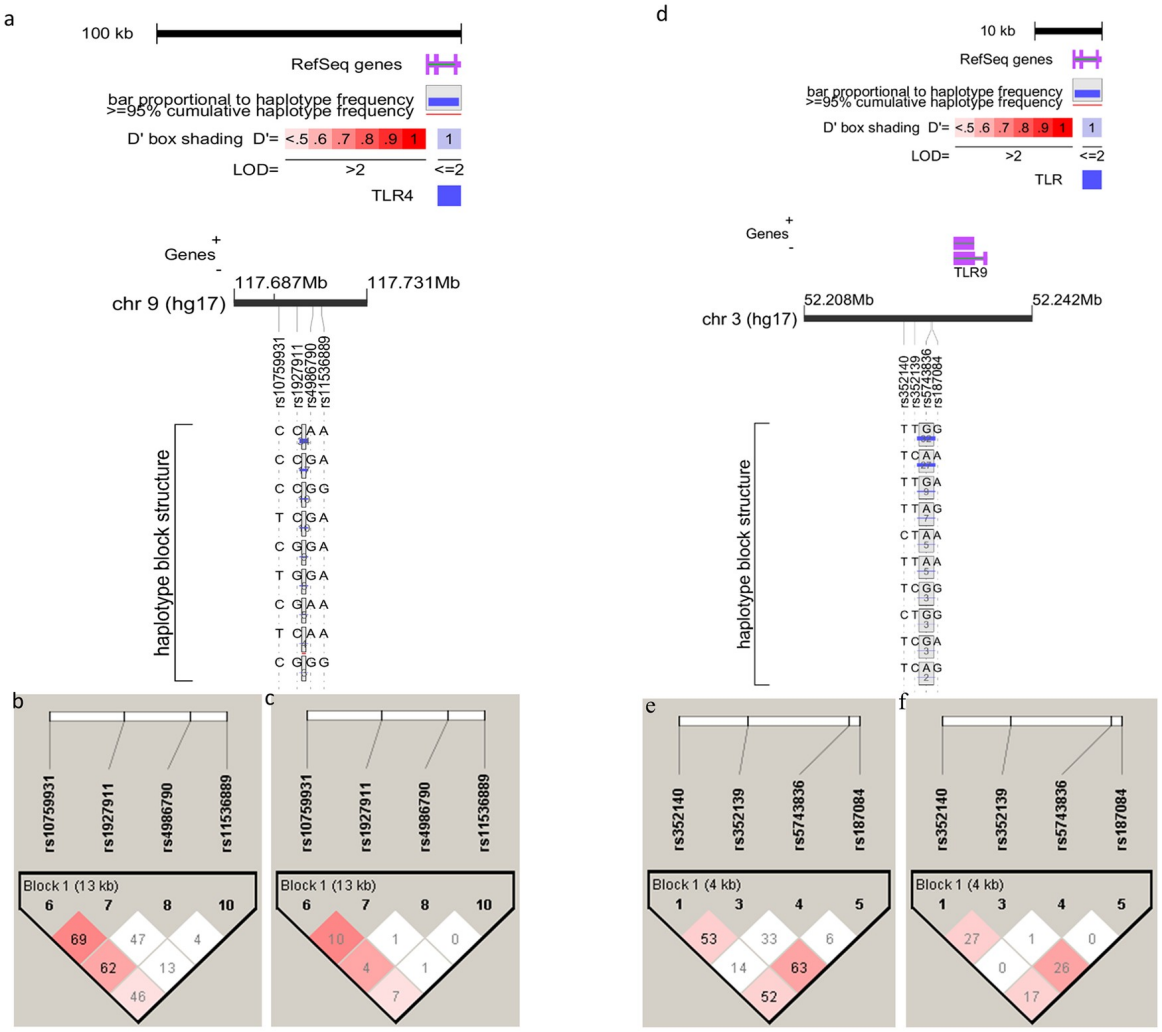
*TLR4* and *TLR9* haplotype block structures, linkage disequilibrium plots and r^2^ plots generated using Haploview and Locusview programs. **a** and **d** represents haplotype block structures, **b** and **e** shows linkage disequilibrium plots, representing the degree of linkage disequilibrium between two SNPs, indicated by the level of pair-wise D’ values shown in the blocks. **c** and **e** represents the r^2^ values with percentage correlation between the two SNPs shown in each box.

Linkage disequilibrium (LD) analysis revealed two SNPs each in *TLR4* (rs1927911; rs10759931), and *TLR9* (rs352139; rs187084) genes were in strong LD. Haplotype blocks were generated using 4 and 3 SNPs of both *TLR4* and *TLR9* genes. The haplotype blocks showing the D’ and r^2^ values, as well as the block structures of *TLR4* and *TLR9* SNPs, are shown in Fig 1.

Six *TLR4* haplotypes (frequency >5%) generated using all the four *TLR4* SNPs showed an accumulated frequency of 84% and 87% in cases and controls respectively, revealing a significant distribution of haplotypes (Pglobal = 0.008) (S4 Table and Fig 1). Additionally, four haplotypes (frequency >5%) were also generated by excluding one *TLR4* SNP at a time. After removing SNP rs10759931, an accumulated frequency of 86.7% and 89.6% of haplotypes was found in cases and controls respectively that showed a significant distribution (Pglobal = 0.0045). Haplotype GCA was found at a significant lower frequency in cases as compared to controls (OR, 0.6 [95% CI, 0.38–0.95]; *p =* 0.0272) (S4 Table and S1 Fig). None of the *TLR4* haplotypes was found to be associated with TV infection (S5 Table). Within cervicitis cases, haplotype AGC obtained after excluding SNP rs11536889 was found to be significantly (OR, 1.87 [95% CI, 1.0–3.53]; *p =* 0.0493) (S6 Table and S1 Fig) associated with increased risk for TV induced cervicitis, when the comparison was made between TV positive and negative patients. No other *TLR4* haplotype revealed significant distribution among cases and controls.

Similarly, in the case of *TLR9* haplotypes (frequency >3%), the six haplotypes generated using all *TLR9* SNPs showed an accumulated frequency of 84% in both cases and controls revealing a non-significant distribution (Pglobal = 0.339) (S7 Table and Fig 1). By excluding SNP rs187084, four *TLR9* haplotypes (frequency >5%) with an accumulated frequency of 84.5% and 88.5% in cases and controls respectively, showed a non-significant distribution (Pglobal = 0.0949) (S7 Table and S2 Fig). Haplotype GTA showed a significantly high occurrence in the cases as compared to controls (OR, 1.99 [95% CI 1.14–3.48]; *p =* 0.014) (S7 Table and S2 Fig). None of the other *TLR9* haplotypes showed significant distribution between cases and controls as well as between TV positive and negative cases within the cervicitis patients (S7–S9 Tables). S4–S9 Tables showing the haplotype frequencies of *TLR4* and *TLR9* SNPs as well as S1 and S2 Figs are provided as Supplementary Data.

## Discussion

Inflammation of the cervix is one of the major health issues of women globally known to be caused by the infection of various pathogens including *C*. *trachomatis*, *N*. *gonorrhoeae*, *T*. *vaginalis* and HPV; wherein TLR4 and TLR9 are known to play a crucial role in the induction of inflammatory response against these pathogens [1,12,13]. The present study, which is the first of its kind, was designed to investigate the role of *TLR4* and *TLR9* SNPs and haplotypes in the susceptibility to abovementioned pathogens and cervicitis.

The present group of cervicitis patients belonged to the western part of India where TV infection was detected in almost one-third of the cases whereas the frequency of CT (1.5%) and NG (2.3%) infection together was less than 4%. This suggests a nongonococal nonchlamydial origin of cervicitis. Previous studies have reported the prevalence of CT and NG from as low as 4% [45] and 1% [46] respectively to as high as 54% [47] and 23.8% [48] respectively. On the other hand, the prevalence of TV has also shown similar variance ranging from nil [45] to 38.4% [48] in cervicitis patients. It has been reported that the consistent presence of TV increases the risk of acquiring HPV infection and thereby augmenting the risk of cervical cancer development [49,50].

We have also observed a low frequency of HPV infection (HPV [4.6%]; HPV16 [2.3%]; HPV18 [0%]) and no coexistence of the investigated pathogens in the cervicitis patients. The prevalence of HPV in cervicitis has shown an inconsistent pattern world-over, as observed in Korea (HPV [56.8%]; HPV16 [10.6%]; HPV18 [6.8%)]) [51], Iran (HPV [98.7%]) [52] and Greece (HPV [36%]; HPV16 [12%]; HPV18 [15%]) [53]. Although our data suggest no significant involvement of HPV in cervicitis; however, women when presented with cervicitis, should also be investigated for the presence of HPV, as the presence of HPV in cervicitis may serve as a compounding factor towards cervical carcinogenesis. Nevertheless, the difference in the prevalence of above-mentioned pathogens in the various studies may be linked to variation in sample size and methodology, and most importantly the background of the study subjects, as the analyzed pathogens are hygiene associated which can be related to the socio-economic status of the participants.

Although no reports are available on the association of *TLR* SNPs and haplotypes with cervicitis, our analysis partially corroborates with previous findings on the diseases associated with pathogen infection and inflammation. We found *TLR4* 3′ UTR rs11536889 CC genotype and C allele to be associated with increased the risk of cervicitis. This polymorphism was associated with gastric atrophy [54], hepatitis B virus recurrence post liver transplantation [55] and peridontitis. On the other hand *TLR4* rs10759931 AG genotype showed a protective effect against cervicitis in our study subjects. However, the same genotype was related to an increased number of inflammatory cells in the sputum of chronic obstructive pulmonary disease patients [56].

On the other hand, we did not find either *TLR4* rs4986790, a synonymous (Asp299Gly) (AG, p = 0.472; GG, p = 0.821) or intronic rs1927911 (CT, p = 0.794; TT, p = 0.861) polymorphism to be associated with cervicitis. The Asp299Gly change was associated with inflammatory bowel diseases [57]. In the case of *TLR4* intronic SNP, rs1927911 has been reported to increase the risk of diabetic foot ulcers [58] and atherosclerotic cerebral infarction [16].

With regard to *TLR9* gene polymorphisms, we found promoter rs187084 TC genotype to be associated with an increased risk of cervicitis. A similar result was obtained in inflammatory bowel diseases [59]. On the contrary, none of the genotypes or alleles of *TLR9* rs5743844, rs352140, rs5743836 or rs352139 polymorphisms were associated with cervicitis. A complete absence of *TLR9* non-synonymous polymorphism rs5743844 (Pro99Leu) in our study population corroborates with the report of Lee and group (2006) where neither controls nor lung disease patients carried the same polymorphism [60]. However, synonymous rs352140 (G2848A/ Pro545Pro) polymorphism was found associated with systemic lupus erythematosus, promoter SNP rs5743836 with asthma, Crohn’s disease and renal disease, and intronic SNP rs352139 with IgA Nephropathy [61]. The disparity of results in studies mentioned above may be linked to differences in sample size, study design and methods of SNP detection, and interethnic variations of the study population.

As haplotypes are considered more informative than SNPs [62], we generated haplotypes from different combinations of *TLR4* and *TLR9* SNPs. *TLR4* GCA and *TLR9* GTA haplotypes were significantly associated with decreased and increased risk of cervicitis respectively. Moreover, within cervicitis cases, haplotype AGC was found to be significantly associated with TV induced cervicitis. Our results indicate that two SNPs each in both *TLR4* (rs1927911; rs10759931), and *TLR9* (rs352139; rs187084) genes were in strong LD. Furthermore, certain SNP pairs in our study deviated from the norm that the linkage disequilibrium is a function of distance, which is accordance with the observations of Stephens et al. (2001) [62]. The *TLR4* SNPs (rs10759931 and rs4986790) that were separated by a distance of 11.1 kb showed strong LD (D′ = 0.62) while the SNPs (rs4986790 and rs11536889) that were separated by a shorter 2.8 kb distance did not exhibit a strong linkage disequilibrium (D′ = 0.04). The SNP pairs rs352140:rs187084 and rs5743836:rs187084 of *TLR9* gene were also in agreement with the above-mentioned trend, where the SNP pair rs352140:rs187084 that were separated by a larger distance (4.3kb) exhibited a stronger LD (D′ = 0.52) as compared to the pair (rs5743836:rs187084) that were separated by a comparatively smaller distance (distance = 0.24kb, D′ = 0.06). S10 Table shows the SNP pairs of *TLR4* and *TLR9* gene, the genetic distance between SNPs and the Dˈ values.

Coming back to pathogen infection and *TLR* polymorphisms, we found *TLR4* rs11536889 CC genotype to be significantly associated with higher risk of TV induced cervicitis. None of the other *TLR4* and *TLR9* SNPs or haplotypes showed association with TV infection. No research group has yet investigated the role of *TLR* polymorphisms in TV induced cervicitis. However, Chen et al., (2013) observed a marginal association of the *TLR4* rs4986790 AG genotype with TV infected prostate cancer patients. In the case of CT, NG, and HPV infections and their association with *TLR* SNPs, limited reports are available worldwide, and none is available on cervicitis. Several reports demonstrate that *TLR4* and *TLR9* polymorphism are associated with CT (***TLR4***: rs1927911 with PID; rs4986790 with PID, tubal pathology and genital tract infections; rs4986791 with genital tract infections; ***TLR9***: rs5743836 and rs352140 with tubal pathology; rs5743836 with CT associated symptoms) and NG (***TLR4***: rs1927911 and rs4986790 with PID) infections and disease association [19,26–29]. On the other hand, Oliveira et al. (2013), failed to identify the association of *TLR9* promoter rs5743836 polymorphism with HPV clearance or persistence healthy women [32]. Due to the statistically low occurrence of CT (1.5%), NG (2.3%) and HPV (4.6%) in our study subjects, no statistical analysis could be performed with either *TLR4* or *TLR9* SNPs and haplotypes. Moreover, exploring the effect of above said polymorphisms on the expression pattern of *TLR4* and *TLR9* genes could provide more insights on the influence of CT, NG, TV, HPV infections on cytokine production and the host immune response.

Based on our results, we suggest a significant influence of *TLR4* and *TLR9* polymorphisms on cervicitis. However, our study also suffered from many limitations. For example, being a hospital-based case-control study, the selection bias could not be excluded. Study on the expression pattern of the TLR4 and TLR9 would have reflected the effect of SNPs. Last but not the least, HPV16 and 18 copy number analysis could have also revealed a probable link between *TLR4* and *TLR9* polymorphisms and their effect of severity of HPV infection.

Cervicitis is though curable using antibiotic regime [63], it is well known that persistent cervicitis is one of the risk factors of cervical carcinogenesis [64,65]. Our study revealed a higher prevalence of TV infection in cervicitis compared to CT, NG, and HPV. The *TLR4* and *TLR9* SNPs as well as haplotypes modulated the cervicitis risk as a whole and TV induced cervicitis as well. Furthermore, elucidation of the functional role of these polymorphisms may help in understanding the pathophysiology of cervicitis. Our results provide lead-in information to develop personalized clinical marker that could be utilised in future as a screening tool. This may be useful in providing primary preventive care by identifying women at greater risk of cervicitis and possibly cervical cancer. Finally, a comprehensive multicentric study on large and varied ethnic populations will help in precisely understanding the clinical relevance and overall impact of both the genes to CT, NG, TV, HPV infections and cervicitis risk.

## Supporting information

S1 Fig *TLR4* haplotype block structures, linkage disequilibrium plots and r^2^ plots generated using Haploview and Locusview programs. **a** and **d** represents haplotype block structures generated excluding rs10759931 and rs11536889 respectively. **b** and **e** shows linkage disequilibrium plots generated excluding rs10759931 and rs11536889 respectively, representing the degree of linkage disequilibrium between two SNPs, indicated by the level of pair-wise D’ values shown in the blocks. **c** and **f** represents the r^2^ values generated excluding rs10759931 with percentage correlation between the two SNPs shown in each box.
(TIF)

S2 Fig *TLR9* haplotype block structures, linkage disequilibrium plots and r^2^ plots generated using Haploview and Locusview programs. **a** and **d** represents haplotype block structures generated excluding rs187084 and rs5743836; respectively. **b** and **e** shows linkage disequilibrium plots generated excluding rs187084 and rs5743836 respectively, representing the degree of linkage disequilibrium between two SNPs, indicated by the level of pair-wise D’ values shown in the blocks. **c** and **f** represents the r^2^ values generated excluding rs187084 and rs5743836 respectively, with percentage correlation between the two SNPs shown in each box.
(TIF)

S1 Table Details of the SNPs included in the study. (DOCX)

S2 Table *TLR4* and *TLR9* PCR primer sequences, thermal profiles and amplicon size. (DOCX)

S3 Table Details of restriction enzymes and accessory information. (DOCX)

S4 Table Distribution of *TLR4* haplotypes in cervicitis patients and controls. (DOCX)

S5 Table *TLR4* haplotypes and the risk for *T*. *vaginalis* infected cervicitis. (DOCX)

S6 Table *TLR4* haplotypes and the risk for *T*. *vaginalis* infected cervicitis within samples. (DOCX)

S7 Table *TLR9* haplotypes and the risk for cervicitis >3%. (DOCX)

S8 Table *TLR9* SNPs haplotypes and the risk for *T*. *vaginalis* infected cervicitis. (DOCX)

S9 Table *TLR9* SNPs haplotypes and the risk for *T*. *vaginalis* infected cervicitis within samples. (DOCX)

S10 Table *TLR4* and *TLR9* SNP pairs, genetic distance between SNPs and corresponding D′ values. (DOCX)

S1 Dataset Raw data of the study. (XLSX)
