# Supplementary figures and images for: Association of TLR4 and TLR9 gene polymorphisms and haplotypes with cervicitis susceptibility
Source: PLoS One. 2019 Jul 31;14(7):e0220330. doi: 10.1371/journal.pone.0220330 (PMC6668796; doi:10.1371/journal.pone.0220330)

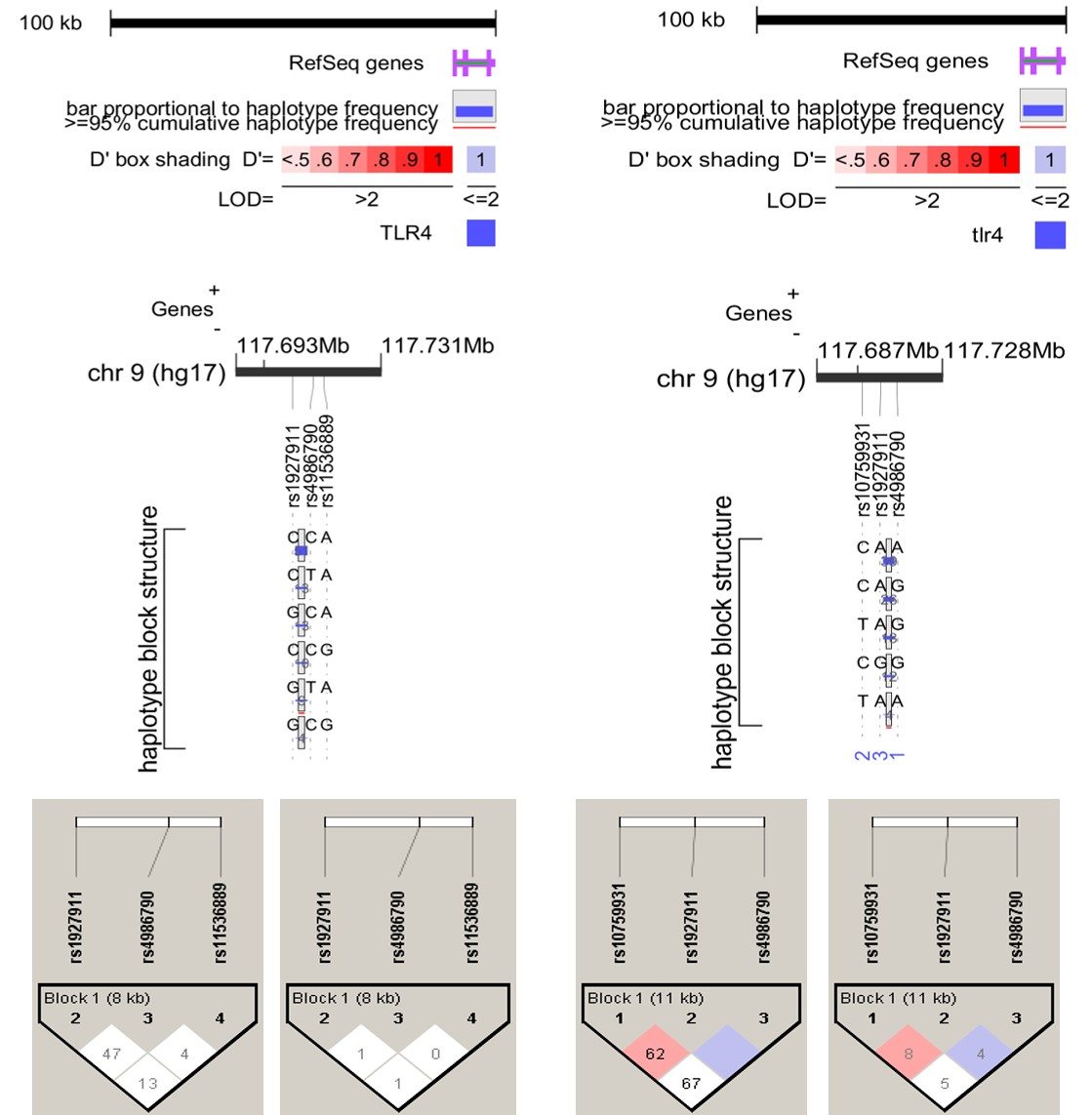

Supplement: S1 Fig — a and d represents haplotype block structures generated excluding rs10759931 and rs11536889 respectively. b and e shows linkage disequilibrium plots generated excluding rs10759931 and rs11536889 respectively, representing the degree of linkage disequilibrium between two SNPs, indicated by the level of pair-wise D’ values shown in the blocks. c and f represents the r2 values generated excluding rs10759931 with percentage correlation between the two SNPs shown in each box. (TIF) [file pone.0220330.s001.tif]

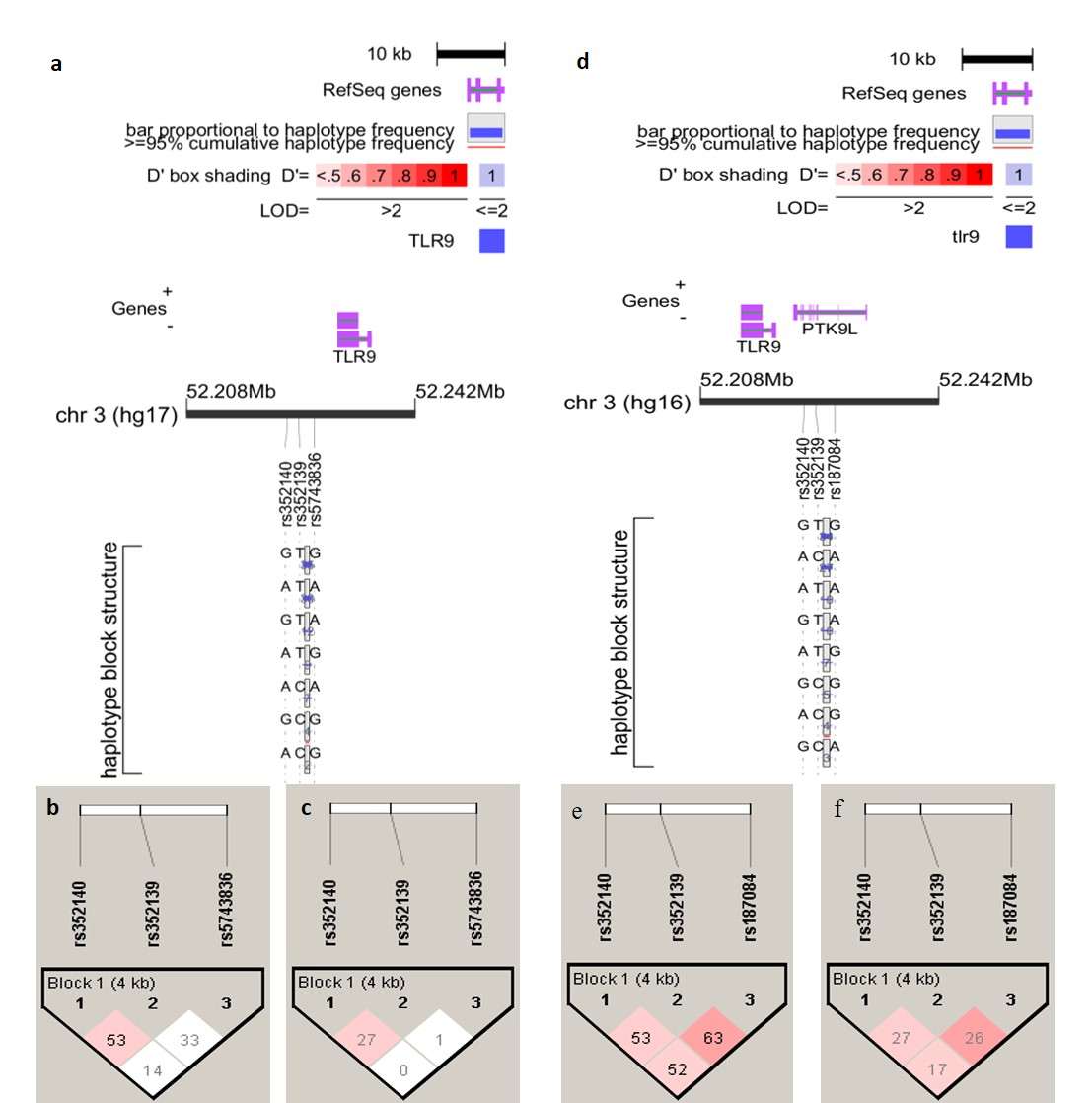

Supplement: S2 Fig — a and d represents haplotype block structures generated excluding rs187084 and rs5743836; respectively. b and e shows linkage disequilibrium plots generated excluding rs187084 and rs5743836 respectively, representing the degree of linkage disequilibrium between two SNPs, indicated by the level of pair-wise D’ values shown in the blocks. c and f represents the r2 values generated excluding rs187084 and rs5743836 respectively, with percentage correlation between the two SNPs shown in each box. (TIF) [file pone.0220330.s002.tif]
