# Supplementary material for: Association of TLR4 and TLR9 gene polymorphisms and haplotypes with cervicitis susceptibility
Source: PLoS One. 2019 Jul 31;14(7):e0220330. doi: 10.1371/journal.pone.0220330 (PMC6668796; doi:10.1371/journal.pone.0220330)
Supplement: S1 Table — (DOCX) [file pone.0220330.s003.docx]

**S1 Table** Details of the SNPs included in the study.

| **Gene** | **rsID** | **SNP** | **AA/Codon Change** | **Location** | **Minor Allele Frequency (MAF)** |
| --- | --- | --- | --- | --- | --- |
| *TLR4* | rs4986790 | 896A>G Asp299Gly | GAT→GGT D [Asp] ⇒ G [Gly] | Exon 3 | 0.0599 |
|  | rs4986791 | 1196C>T (Thr399Ile) | ACC→ATC T [Thr] ⇒ I [Ile] | Exon 3 | 0.0407 |
|  | rs10759931 | 2688A>G | NA | 5' Near Gene | 0.3514 |
|  | rs11536889 | 3725G>C | NA | Exon 4 | 0.1378 |
|  | rs1927911 | 7764C>T | NA | Intron 1 | 0.4002 |
| *TLR9* | rs187084 | -1486T>C | NA | 5' UTR | 0.3776 |
|  | rs5743836 | -1237T>C | NA | 5' UTR | 0.1725 |
|  | rs352140 | 2848G>A | CCG→CCA P [Pro] ⇒ P [Pro] | Exon 2 | 0.4155 |
|  | rs352139 | 1174A>G | NA | Intron 1 | 0.4900 |
|  | rs5743844 | 296C>T | CCG→CTG P [Pro] ⇒ L [Leu] | Exon 2 | 0.002 |
| Abbreviations: *TLR*, Toll-like receptor; rsID, reference sequence ID; SNP, single nucleotide polymorphism. | | | | |  |
