# Supplementary material for: Association of TLR4 and TLR9 gene polymorphisms and haplotypes with cervicitis susceptibility
Source: PLoS One. 2019 Jul 31;14(7):e0220330. doi: 10.1371/journal.pone.0220330 (PMC6668796; doi:10.1371/journal.pone.0220330)
Supplement: S2 Table — (DOCX) [file pone.0220330.s004.docx]

**S2** **Table** *TLR4* and *TLR9* PCR primer sequences, thermal profiles and amplicon size

| **rsID** | **Primers (5’ – 3’)** | **Thermal Profile** | **Amplicon (bp)** | **Ref.** |
| --- | --- | --- | --- | --- |
| rs4986790 | F: GATTAGCATACTTAGACTACTACCTCCATG  R: GATCAACTTCTGAAAAAGCATTCCCAC | (95°-5')1 (94°-40'', 55°-40'', 72°-60'')36 (72°-10')1 | 249 | [1] |
| rs4986791 | F: GGTTGCTGTTCTCAAAGTGATTTTGGGAGAA  R:ACCTGAAGACTGGAGAGTGAGTTAAATGCT | (95°-5')1 (94°-40'', 60°-40'', 72°-60'')36 (72°-10')1 | 406 | [1] |
| rs10759931 | F: ATAACCTCAGTGGGCTCTGG  R: ATGTTCTGGCATCTGGGAAG | (94°-5')1 (94°-40'', 58°-45'', 72°-40'')35 (72°-10')1 | 241 | [2] |
| rs11536889 | F: ACAAGTGATGTTTGATGGAC  R: GCCATTCTACCTGGTATAAG | (94°-6')1 (94°-60'', 55°-60'', 72°-2’)35 (72°-10')1 | 203 | [2] |
| rs1927911 | F :TCACTTTGCTCAAGGGTCAA  R: AAACCTGCATGCTCTGCAC | (94°-5')1 (94°-40'', 58°-45'', 72°-40'')35 (72°-10')1 | 361 | [3] |
| rs187084 | F: TCCCAGCAGCAACAATTCATTA  R: CTGCTTGCAGTTGACTGTGT | (95°-5')1 (95°-40'', 60°-40'', 72°-60'')36 (72°-10')1 | 499 | [1] |
| rs5743836 | F: ATGGGAGCAGAGACATAATGGA  R: CTGCTTGCACTTGACTGTGT | (95°-5')1 (94°-40'', 62°-40'', 72°-60'')35 (72°-10')1 | 135 | [1] |
| rs352140 | F: AAGCTGGACCTCTACCACGA  R: TTGGCTGTGGATGTTGTT | (95°-5')1 (94°-45'', 56°-60'', 72°-30'')35 (72°-10')1 | 177 | [4] |
| rs352139 | AFP: AAGTGGAGTGGGTGGAGGTA  GFP: GTGGAGTGGGTGGAGGTG  R: CAAGGAAAGGCTGGTGACAT | (95°-5')1 (94°-60'', 64°-60'', 72°-60'')35 (72°-4')1 | 270 | [5] |
| rs5743844 | F: GGATGTTGGTATGGCTGAGG  R: AACTGCAACTGGCTGTTCCT | (95°-5')1 (94°-45'', 56°-60'', 72°-30'')35 (72°-10')1 | 337 | [6] |
| Abbreviations: rsID, reference sequence ID; bp, base pairs. | | | |  |

**References**

1. Liu F, Lu W, Qian Q, et al (2012) Frequency of TLR 2, 4, and 9 gene polymorphisms in Chinese population and their susceptibility to type 2 diabetes and coronary artery disease. J Biomed Biotechnol 2012:. https://doi.org/10.1155/2012/373945

2. Singh K, Singh K, Singh VK, et al (2013) Association of toll-like receptor 4 polymorphisms with diabetic foot ulcers and application of artificial neural network in DFU risk assessment in type 2 diabetes patients. Biomed Res Int 2013:. https://doi.org/10.1155/2013/318686

3. Shen Y, Liu Y, Liu S, Zhang A (2013) Toll-like Receptor 4 Gene Polymorphisms and Susceptibility to Bladder Cancer. Pathol Oncol Res 19:275–280. https://doi.org/10.1007/s12253-012-9579-8

4. Pandey S, Mittal B, Srivastava M, et al (2011) Evaluation of Toll-like receptors 3 (c.1377C/T) and 9 (G2848A) gene polymorphisms in cervical cancer susceptibility. Mol Biol Rep 38:4715–4721. https://doi.org/10.1007/s11033-010-0607-z

5. Shahin RMH, El Khateeb E, Khalifa RH, El Refai RM (2016) Contribution of toll-like receptor 9 gene single-nucleotide polymorphism to systemic lupus erythematosus in Egyptian patients. Immunol Invest 45:235–242. https://doi.org/10.3109/08820139.2015.1137934

6. Kubarenko A V., Ranjan S, Rautanen A, et al (2010) A naturally occurring variant in human TLR9, P99L, is associated with loss of CpG oligonucleotide responsiveness. J Biol Chem 285:36486–36494. https://doi.org/10.1074/jbc.M110.117200
