# Supplementary material for: Association of TLR4 and TLR9 gene polymorphisms and haplotypes with cervicitis susceptibility
Source: PLoS One. 2019 Jul 31;14(7):e0220330. doi: 10.1371/journal.pone.0220330 (PMC6668796; doi:10.1371/journal.pone.0220330)
Supplement: S3 Table — (DOCX) [file pone.0220330.s005.docx]

**S3** **Table** Details of Restriction enzymes and accessory information

| **rsID** | **Restriction Enzyme** | **Product size and genotypes (bp)** | **Visualized on** | **Reference** |
| --- | --- | --- | --- | --- |
| rs4986790 | *NcoI* | 249 = AA 249, 223, 26 = AG 223, 26 = GG | 15% PAGE | [1] |
| rs4986791 | *HinfI* | 406 = CC 406, 377, 29 = CT 377, 29 = TT | 15% PAGE | [1] |
| rs10759931 | *KpnI* | 241 = AA  241, 190, 51 = AG  190, 51 = GG | 2.5% Agarose | [2] |
| rs1927911 | *StyI* | 203 = TT 203, 178, 25 = TC 178, 25 = CC | 15% PAGE | [2] |
| rs11536889 | *EarI* | 361 = CC 361, 198, 163 = CG 198, 163 = GG | 2% Agarose | [3] |
| rs187084 | *AflII* | 499 = CC 499, 327, 172 = CT 327, 172 = TT | 2% Agarose | [1] |
| rs5743836 | *BstNI* | 108, 27 = TT 108, 60, 48, 27 = TC 60, 48, 27 = CC | 15% PAGE | [1] |
| rs352140 | *BstUI* | 177 = AA 177, 135, 42 = AG 135, 42 = GG | 12% PAGE | [4] |
| rs5743844 | *BslI* | 203, 134 = TT 203, 168, 134, 35 = TC  168, 134, 35 = CC | 15% PAGE | [6] |
| Abbreviations: rsID, reference sequence ID; bp, base pairs. | | | | |

**References**

1. Liu F, Lu W, Qian Q, et al (2012) Frequency of TLR 2, 4, and 9 gene polymorphisms in Chinese population and their susceptibility to type 2 diabetes and coronary artery disease. J Biomed Biotechnol 2012:. https://doi.org/10.1155/2012/373945

2. Singh K, Singh K, Singh VK, et al (2013) Association of toll-like receptor 4 polymorphisms with diabetic foot ulcers and application of artificial neural network in DFU risk assessment in type 2 diabetes patients. Biomed Res Int 2013:. https://doi.org/10.1155/2013/318686

3. Shen Y, Liu Y, Liu S, Zhang A (2013) Toll-like Receptor 4 Gene Polymorphisms and Susceptibility to Bladder Cancer. Pathol Oncol Res 19:275–280. https://doi.org/10.1007/s12253-012-9579-8

4. Pandey S, Mittal B, Srivastava M, et al (2011) Evaluation of Toll-like receptors 3 (c.1377C/T) and 9 (G2848A) gene polymorphisms in cervical cancer susceptibility. Mol Biol Rep 38:4715–4721. https://doi.org/10.1007/s11033-010-0607-z

5. Shahin RMH, El Khateeb E, Khalifa RH, El Refai RM (2016) Contribution of toll-like receptor 9 gene single-nucleotide polymorphism to systemic lupus erythematosus in Egyptian patients. Immunol Invest 45:235–242. https://doi.org/10.3109/08820139.2015.1137934

6. Kubarenko A V., Ranjan S, Rautanen A, et al (2010) A naturally occurring variant in human TLR9, P99L, is associated with loss of CpG oligonucleotide responsiveness. J Biol Chem 285:36486–36494. https://doi.org/10.1074/jbc.M110.117200
