# Supplementary material for: Association of TLR4 and TLR9 gene polymorphisms and haplotypes with cervicitis susceptibility
Source: PLoS One. 2019 Jul 31;14(7):e0220330. doi: 10.1371/journal.pone.0220330 (PMC6668796; doi:10.1371/journal.pone.0220330)
Supplement: S4 Table — (DOCX) [file pone.0220330.s006.docx]

**S4** **Table** Distribution of *TLR4* haplotypes in cervicitis patients and controls

| **Haplotype** | **Frequency** | | **OR**  **(95% CI)** | **Global**  ***p*-value** | ***p*-value** |
| --- | --- | --- | --- | --- | --- |
|  | **Cases** | **Controls** |  |  |  |
|  |  |  |  | **0.008** |  |
| CCAA | 30.5 | 33.5 | 1.15 (0.79 – 2.75) |  | 0.4655 |
| CCGA | 13.7 | 16.3 | 1.22 (0.75 – 1.99) |  | 0.4309 |
| CCGG | 6.7 | 9.4 | 1.44 (0.75 – 2.75) |  | 0.2624 |
| TCGA | 9.5 | 11.0 | 1.19 (0.67 – 2.12) |  | 0.5612 |
| CGGA | 14.4 | 9.8 | 0.65 (0.37 – 1.13) |  | 0.1226 |
| TGGA | 9.3 | 7.0 | 0.74 (0.38 – 1.41) |  | 0.3525 |
| **Excluding SNP rs10759931** | | | | | |
|  |  |  |  | **0.0045** |  |
| CCA | 44.0 | 50.3 | 1.28 (0.90 – 1.83) |  | 0.1634 |
| GCA | 22.8 | 15.0 | 0.6 (0.38 – 0.95) |  | **0.0272** |
| CTA | 12.0 | 13.9 | 1.18 (0.70 – 1.99) |  | 0.5366 |
| CCG | 7.9 | 10.4 | 1.36 (0.74 – 2.50) |  | 0.3224 |
| ***Excluding SNP rs11536889*** | | | | | |
|  |  |  |  | *0.541* |  |
| AAC | 38.6 | 38.0 | 01.02 (0.71 – 1.47) |  | 0.8945 |
| AGC | 26.3 | 28.2 | 0.91 (0.61 – 1.35) |  | 0.6347 |
| AGT | 18.0 | 18.8 | 0.95 (0.6 – 1.5) |  | 0.8316 |
| GGC | 11.4 | 9.3 | 1.26 (0.71 – 2.24) |  | 0.4302 |
| Global *p*-values as well as *p*-values were calculated using FAMHAP. *p*<0.05 were considered statistically significant. Significant values are represented in bold.  Abbreviations: *TLR*, Toll-like receptor; OR, odds ratio; CI, confidence interval | | | | | |
