# Supplementary material for: Association of TLR4 and TLR9 gene polymorphisms and haplotypes with cervicitis susceptibility
Source: PLoS One. 2019 Jul 31;14(7):e0220330. doi: 10.1371/journal.pone.0220330 (PMC6668796; doi:10.1371/journal.pone.0220330)
Supplement: S5 Table — (DOCX) [file pone.0220330.s007.docx]

**S5** **Table** *TLR4* haplotypes and the risk for *T. vaginalis* infected cervicitis

| **Haplotype** | **Frequency** | | **OR**  **(95% CI)** | **Global**  ***p*-value** | ***p*-value** |
| --- | --- | --- | --- | --- | --- |
|  | **Cases** | **Controls** |  |  |  |
|  |  |  |  | 0.423 |  |
| CCAA | 31.1 | 26.6 | 0.80 (0.44 – 1.47) |  | 0.4796 |
| CCGA | 13.9 | 21.6 | 1.71 (0.87 – 3.37) |  | 0.1161 |
| GCGA | 14.3 | 13.9 | 0.97 (0.45 – 2.09) |  | 0.9228 |
| TGGA | 9.7 | 8.3 | 0.85 (0.33 – 2.2) |  | 0.7314 |
| TCGA | 8.7 | 7.3 | 0.82 (0.3 – 2.28) |  | 0.7188 |
| CGCG | 6.8 | 11.1 | 1.69 (0.69 – 4.12) |  | 0.2399 |
| **Excluding SNP rs10759931** | | | | | |
|  |  |  |  | 0.404 |  |
| CCA | 44.2 | 48.5 | 1.19 (0.7 – 2.04) |  | 0.5232 |
| GCA | 22.9 | 16.7 | 0.67 (0.33 – 1.36) |  | 0.2686 |
| CTA | 12.0 | 11.1 | 0.91 (0.39 – 2.13) |  | 0.8322 |
| CCG | 8.0 | 11.6 | 1.52 (0.64 – 3.61) |  | 0.3459 |
| ***Excluding SNP rs11536889*** | | | | | |
|  |  |  |  | *0.405* |  |
| AAC | 28.8 | 38.3 | 0.65 (0.36 – 1.17) |  | 0.149 |
| AGC | 35.5 | 28.3 | 1.39 (0.79 – 2.45) |  | 0.2546 |
| AGT | 15.6 | 18.2 | 0.83 (0.4 – 1.73) |  | 0.6204 |
| GGC | 15.0 | 9.2 | 1.74 (0.79 – 3.83) |  | 0.1624 |
| Global *p*-values as well as *p*-values were calculated using FAMHAP. *p*<0.05 were considered statistically significant. Significant values are represented in bold.  Abbreviations: *TLR*, Toll-like receptor; OR, odds ratio; CI, confidence interval | | | | | |
