# Supplementary material for: Association of TLR4 and TLR9 gene polymorphisms and haplotypes with cervicitis susceptibility
Source: PLoS One. 2019 Jul 31;14(7):e0220330. doi: 10.1371/journal.pone.0220330 (PMC6668796; doi:10.1371/journal.pone.0220330)
Supplement: S6 Table — (DOCX) [file pone.0220330.s008.docx]

**S6** **Table** *TLR4* haplotypes and the risk for *T. vaginalis* infected cervicitis within samples

| **Haplotype** | **Frequency** | | **OR**  **(95% CI)** | **Global**  ***p*-value** | ***p*-value** |
| --- | --- | --- | --- | --- | --- |
|  | **Cases** | **Controls** |  |  |  |
|  |  |  |  | 0.32 |  |
| CCAA | 36.7 | 27.0 | 0.64 (0.34 – 1.2) |  | 0.1616 |
| CCGA | 15.1 | 22.3 | 1.61 (0.78 – 3.33) |  | 0.1959 |
| CCGG | 9.2 | 11.8 | 1.32 (0.52 – 3.32) |  | 0.5545 |
| TCGA | 11.0 | 6.6 | 0.58 (0.19 – 1.71) |  | 0.3136 |
| CGGA | 7.0 | 12.3 | 1.86 (0.72 – 4.83) |  | 0.1944 |
| TGGA | 7.8 | 9.5 | 1.24 (0.45 – 3.39) |  | 0.6761 |
| **Excluding SNP rs10759931** | | | | | |
|  |  |  |  | 0.821 |  |
| CCA | 51.9 | 50.1 | 0.93 (0.52 – 1.65) |  | 0.8035 |
| CTA | 14.3 | 9.9 | 0.66 (0.26 – 1.67) |  | 0.3824 |
| GCA | 12.6 | 13.5 | 1.08 (0.46 – 2.53) |  | 0.8543 |
| CCG | 9.8 | 11.2 | 1.16 (0.46 – 2.94) |  | 0.7539 |
| ***Excluding SNP rs11536889*** | | | | | |
|  |  |  |  | *0.174* |  |
| AAC | 29.0 | 42.6 | 0.55 (0.3 – 1.03) |  | 0.0583 |
| AGC | 34.6 | 22.0 | 1.87 (1.0 – 3.53) |  | **0.0493** |
| AGT | 16.1 | 19.2 | 0.81 (0.38 – 1.74) |  | 0.5872 |
| GGC | 15.9 | 10.7 | 1.59 (0.69 – 3.64) |  | 0.2736 |
| Global *p*-values as well as *p*-values were calculated using FAMHAP. *p*<0.05 were considered statistically significant. Significant values are represented in bold.  Abbreviations: *TLR*, Toll-like receptor; OR, odds ratio; CI, confidence interval | | | | | |
