# Supplementary material for: Association of TLR4 and TLR9 gene polymorphisms and haplotypes with cervicitis susceptibility
Source: PLoS One. 2019 Jul 31;14(7):e0220330. doi: 10.1371/journal.pone.0220330 (PMC6668796; doi:10.1371/journal.pone.0220330)
Supplement: S7 Table — (DOCX) [file pone.0220330.s009.docx]

**S7** **Table** *TLR9* haplotypes and the risk for cervicitis >3%

| **Haplotype** | **Frequency** | | **OR**  **(95% CI)** | **Global**  ***p*-value** | ***p*-value** |
| --- | --- | --- | --- | --- | --- |
|  | **Cases** | **Controls** |  |  |  |
|  |  |  |  | 0.339 |  |
| TTGG | 33.7 | 28.8 | 0.80 (0.54 – 1.17) |  | 0.2432 |
| TCAA | 25.5 | 27.9 | 1.13 (0.76 – 1.68) |  | 0.5513 |
| TTGA | 7.1 | 11.8 | 1.74 (0.95 – 3.20) |  | 0.0725 |
| TTAG | 6.7 | 6.9 | 1.02 (0.51 – 2.06) |  | 0.9476 |
| CTAA | 5.3 | 4.7 | 0.87 (0.39 – 1.96) |  | 0.739 |
| TTAA | 5.5 | 3.6 | 0.65 (0.27 – 1.56) |  | 0.3358 |
| ***Excluding SNP rs187084*** | | | | | |
|  |  |  |  | *0.0949* |  |
| *GTG* | *38.3* | *31.7* | *0.75 (0.51 – 1.08)* |  | *0.1195* |
| *ATA* | *30.1* | *30.5* | *1.02 (0.69 – 1.49)* |  | *0.9328* |
| *GTA* | *8.3* | *15.4* | *1.99 (1.14 – 3.48)* |  | ***0.014*** |
| *ATG* | *7.8* | *10.9* | *1.44 (0.79 – 2.64)* |  | *0.2374* |
| ***Excluding SNP rs5743836*** | | | | | |
|  |  |  |  | *0.256* |  |
| *GGT* | *30.1* | *37.9* | *0.71 (0.49 – 1.03* |  | ***0.0679*** |
| *AAC* | *27.9* | *26.2* | *1.09 (0.73 – 1.62)* |  | *0.6766* |
| *AAT* | *8.7* | *11,3* | *0.75 (0.41 – 1.35)* |  | *0.3374* |
| *AGT* | *12.1* | *8.0* | *1.59 (0.88 – 2.86)* |  | *0.1212* |
| ***Excluding SNP rs352139*** | | | | | |
|  |  |  |  | *0.717* |  |
| *GTT* | *35.4* | *40.2* | *0.81 (0.57 – 1.17)* |  | *0.2632* |
| *ATC* | *31.1* | *27.0* | *1.22 (0.83 – 1.80)* |  | *0.3146* |
| *ATT* | *15.8* | *12.8* | *1.28 (0.78 – 2.12)* |  | *0.3276* |
| *ACT* | *4.5* | *6.8* | *0.65 (0.30 – 1.43)* |  | *0.2843* |
| ***Excluding SNP rs352140*** | | | | | |
|  |  |  |  | *0.602* |  |
| *GTT* | *40.6* | *40.8* | *0.99 (0.69 – 1.42)* |  | *0.9666* |
| *ATC* | *31.0* | *25.8* | *1.29 (0.87 – 1.90)* |  | *0.2* |
| *ATT* | *10.4* | *12.7* | *0.8 (0.46 – 1.39)* |  | *0.4262* |
| *GTC* | *6.3* | *5.3* | *1.22 (0.57 – 2.57)* |  | *0.6103* |
| Global *p*-values as well as *p*-values were calculated using FAMHAP. *p*<0.05 were considered statistically significant. Significant values are represented in bold.  Abbreviations: *TLR*, Toll-like receptor; OR, odds ratio; CI, confidence interval | | | | | |
