# Supplementary material for: Association of TLR4 and TLR9 gene polymorphisms and haplotypes with cervicitis susceptibility
Source: PLoS One. 2019 Jul 31;14(7):e0220330. doi: 10.1371/journal.pone.0220330 (PMC6668796; doi:10.1371/journal.pone.0220330)
Supplement: S8 Table — (DOCX) [file pone.0220330.s010.docx]

**S8** **Table** *TLR9* SNPs haplotypes and the risk for *T. vaginalis* infected cervicitis

| **Haplotype** | **Frequency** | | **OR**  **(95% CI)** | **Global**  ***p*-value** | ***p*-value** |
| --- | --- | --- | --- | --- | --- |
|  | **Cases** | **Controls** |  |  |  |
|  |  |  |  | 0.707 |  |
| TTGG | 33.9 | 27.9 | 0.76 (0.42 – 1.36) |  | 0.3515 |
| TCAA | 25.5 | 29.7 | 1.24 (0.68 – 2.24) |  | 0.4811 |
| TTGA | 7.3 | 11.6 | 1.67 (0.69 – 4.01) |  | 0.2493 |
| TTAG | 6.3 | 6.2 | 0.97 (0.32 – 2.96) |  | 0.9615 |
| CTAA | 5.2 | 4.5 | 0.86 (0.24 – 3.07) |  | 0.8102 |
| TTAA | 5.8 | 3.5 | 0.58 (0.14 – 2.39) |  | 0.447 |
| ***Excluding SNP rs187084*** | | | | | |
|  |  |  |  | *0.531* |  |
| *GTG* | *38.8* | *30.8* | *0.7 (0.4 – 1.25)* |  | *0.2278* |
| *ATA* | *30.0* | *31.8* | *1.09 (0.61 – 1.94)* |  | *0.7777* |
| *GTA* | *8.4* | *14.9* | *1.92 (0.86 – 4.26)* |  | *1.055* |
| *ATG* | *7.6* | *13.4* | *1.89 (0.82 – 4.35)* |  | *0.1295* |
| ***Excluding SNP rs5743836*** | | | | | |
|  |  |  |  | *0.558* |  |
| *GGT* | *27.9* | *37.9* | *0.63 (0.35 – 1.14)* |  | *0.1265* |
| *AAC* | *29.6* | *26.1* | *1.19 (0.66 – 2.15)* |  | *0.5672* |
| *AAT* | *8.3* | *11.5* | *0.70 (0.27 – 1.81)* |  | *0.4587* |
| *AGT* | *8.6* | *3.2* | *2.85 (0.96 – 8.46)* |  | ***0.049*** |
| ***Excluding SNP rs352139*** | | | | | |
|  |  |  |  | *0.529* |  |
| *GTT* | *33.6* | *40.0* | *0.76 (0.43 – 1.33)* |  | *0.3369* |
| *ATC* | *32.8* | *26.8* | *1.34 (0.75 – 2.38)* |  | *0.325* |
| *ATT* | *15.5* | *13.3* | *1.20 (0.57 – 2.53)* |  | *0.6368* |
| *ACT* | *4.2* | *6.9* | *0.6 (0.16 – 2.16)* |  | *0.4283* |
| ***Excluding SNP rs352140*** | | | | | |
|  |  |  |  | *0.552* |  |
| *GTT* | *39.5* | *41.2* | *0.93 (0.54 – 1.61)* |  | *0.8002* |
| *ATC* | *35.8* | *25.6* | *1.62 (0.92 – 2.87)* |  | *0.0942* |
| *ATT* | *9.7* | *12.7* | *0.74 (0.30 – 1.79)* |  | *0.4993* |
| *GTC* | *5.9* | *5.3* | *1.13 (0.36 – 3.57)* |  | *0.8311* |
| Global *p*-values as well as *p*-values were calculated using FAMHAP. *p*<0.05 were considered statistically significant. Significant values are represented in bold.  Abbreviations: *TLR*, Toll-like receptor; OR, odds ratio; CI, confidence interval | | | | | |
