# Supplementary material for: Association of TLR4 and TLR9 gene polymorphisms and haplotypes with cervicitis susceptibility
Source: PLoS One. 2019 Jul 31;14(7):e0220330. doi: 10.1371/journal.pone.0220330 (PMC6668796; doi:10.1371/journal.pone.0220330)
Supplement: S9 Table — (DOCX) [file pone.0220330.s011.docx]

**S9** **Table** *TLR9* SNPs haplotypes and the risk for *T. vaginalis* infected cervicitis within samples

| **Haplotype** | **Frequency** | | **OR**  **(95% CI)** | **Global**  ***p*-value** | ***p*-value** |
| --- | --- | --- | --- | --- | --- |
|  | **Cases** | **Controls** |  |  |  |
|  |  |  |  | 0.955 |  |
| TTGG | 29.6 | 27.0 | 0.88 (0.46 – 1.68) |  | 0.6973 |
| TCAA | 26.4 | 29.3 | 1.15 (0.61 – 2.18) |  | 0.6549 |
| TTGA | 12.5 | 13.0 | 1.06 (0.45 – 2.49) |  | 0.9133 |
| TTAG | 6.4 | 6.4 | 1.0 (0.32 – 3.2) |  | 0.9899 |
| CTAA | 4.9 | 4.6 | 0.94 (0.24 – 3.67) |  | 0.9363 |
| GATC | 2.7 | 7.2 | 2.81 (0.73 – 10.81) |  | 0.1191 |
| ***Excluding SNP rs187084*** | | | | | |
|  |  |  |  | *0.899* |  |
| *GTG* | *30.9* | *28.6* | *0.89 (0.48 – 1.68)* |  | *0.7267* |
| *ATA* | *28.4* | *30.1* | *1.08 (0.58 – 2.03)* |  | *0.8017* |
| *GTA* | *17.1* | *17.1* | *1.0 (0.57 – 2.15)* |  | *0.9981* |
| *ATG* | *10.9* | *15.1* | *1.46 (0.63 – 3.37)* |  | *0.3762* |
| ***Excluding SNP rs5743836*** | | | | | |
|  |  |  |  | *0.898* |  |
| *GGT* | *26.0* | *30.4* | *0.82 (0.42 – 1.54)* |  | *0.5102* |
| *AAC* | *28.8* | *26.4* | *1.13 (0.59 – 2.14)* |  | *0.7136* |
| *AGT* | *13.5* | *13.0* | *1.05 (0.45 – 2.44)* |  | *0.9136* |
| *AAT* | *7.0* | *8.7* | *0.80 (0.27 – 2.39)* |  | *0.6885* |
| ***Excluding SNP rs352139*** | | | | | |
|  |  |  |  | *0.881* |  |
| *GTT* | *33.0* | *35.7* | *0.89 (0.49 – 1.63)* |  | *0.6992* |
| *ATC* | *32.4* | *30.0* | *1.12 (0.60 – 2.07)* |  | *0.7222* |
| *ATT* | *16.3* | *16.4* | *0.99 (0.45 – 2.15)* |  | *0.9772* |
| *GTC* | *9.3* | *5.3* | *1.84 (0.62 – 5.45)* |  | *0.2642* |
| ***Excluding SNP rs352140*** | | | | | |
|  |  |  |  | *0.88* |  |
| *GTT* | *40.1* | *41.8* | *0.93 (0.52 – 1.67)* |  | *0.8069* |
| *ATC* | *36.5* | *29.0* | *1.41 (0.77 – 2.58)* |  | *0.2699* |
| *ATT* | *8.8* | *10.1* | *0.86 (0.32 – 2.32)* |  | *0.7606* |
| *GTC* | *5.6* | *6.4* | *0.86 (0.25 – 2.94)* |  | *0.8123* |
| Global *p*-values as well as *p*-values were calculated using FAMHAP. *p*<0.05 were considered statistically significant. Significant values are represented in bold.  Abbreviations: *TLR*, Toll-like receptor; OR, odds ratio; CI, confidence interval | | | | | |
