# Supplementary material for: Association of TLR4 and TLR9 gene polymorphisms and haplotypes with cervicitis susceptibility
Source: PLoS One. 2019 Jul 31;14(7):e0220330. doi: 10.1371/journal.pone.0220330 (PMC6668796; doi:10.1371/journal.pone.0220330)
Supplement: S10 Table — (DOCX) [file pone.0220330.s012.docx]

**S10 Table** *TLR4* and *TLR9* SNP pairs, genetic distance between SNPs and corresponding D′ values.

| **Gene** | **SNP combinations** | **Distance (bp)** | **LD (D′)** |
| --- | --- | --- | --- |
| *TLR4* | rs10759931-rs1927911 | 5907 | 0.69 |
|  | rs10759931-rs4986790 | 11155 | 0.62 |
|  | rs10759931-rs11536889 | 13984 | 0.46 |
|  | rs1927911-rs4986790 | 5248 | 0.47 |
|  | rs1927911-rs11536889 | 8077 | 0.13 |
|  | rs4986790 -rs11536889 | 2829 | 0.04 |
| *TLR9* | rs352140-rs352139 | 1675 | 0.53 |
|  | rs352140-rs5743836 | 4085 | 0.14 |
|  | rs352140-rs187084 | 4334 | 0.52 |
|  | rs352139-rs5743836 | 2410 | 0.33 |
|  | rs352139-rs187084 | 2659 | 0.63 |
|  | rs5743836-rs187084 | 249 | 0.06 |
